# Manual versus mechanical cardiopulmonary resuscitation. An experimental study in pigs

**DOI:** 10.1186/1471-2261-10-53

**Published:** 2010-10-28

**Authors:** Qiuming Liao, Trygve Sjöberg, Audrius Paskevicius, Björn Wohlfart, Stig Steen

**Affiliations:** 1 Department of Cardiothoracic Surgery, Lund University and Skåne University Hospital/Lund, Lund, Sweden

## Abstract

**Background:**

Optimal manual closed chest compressions are difficult to give. A mechanical compression/decompression device, named LUCAS, is programmed to give compression according to the latest international guidelines (2005) for cardiopulmonary resuscitation (CPR). The aim of the present study was to compare manual CPR with LUCAS-CPR.

**Methods:**

30 kg pigs were anesthetized and intubated. After a base-line period and five minutes of ventricular fibrillation, manual CPR (n = 8) or LUCAS-CPR (n = 8) was started and run for 20 minutes. Professional paramedics gave manual chest compression's alternating in 2-minute periods. Ventilation, one breath for each 10 compressions, was given to all animals. Defibrillation and, if needed, adrenaline were given to obtain a return of spontaneous circulation (ROSC).

**Results:**

The mean coronary perfusion pressure was significantly (p < 0.01) higher in the mechanical group, around 20 mmHg, compared to around 5 mmHg in the manual group. In the manual group 54 rib fractures occurred compared to 33 in the LUCAS group (p < 0.01). In the manual group one severe liver injury and one pressure pneumothorax were also seen. All 8 pigs in the mechanical group achieved ROSC, as compared with 3 pigs in the manual group.

**Conclusions:**

LUCAS-CPR gave significantly higher coronary perfusion pressure and significantly fewer rib fractures than manual CPR in this porcine model.

## Background

Studies of cardiopulmonary resuscitation (CPR) events have shown how difficult it is to give optimal chest compressions manually [1-11]. These studies have identified many factors that make manual CPR suboptimal, e.g. rescuer fatigue within 2 minutes, too shallow or too deep chest compressions, too high or too low compression and ventilation rates, too small body size of the rescuer, CPR during transport, especially in stairs or in ambulances, too long pre- and post-shock pauses and too many interruptions in the chest compressions.

In the latest guidelines for CPR from 2005, recommendations to improve the delivery of chest compressions were given [12,13]. To give effective chest compressions, rescuers are advised to "push hard and push fast", at a rate of about 100 compressions per minute. The chest should be allowed to recoil freely after each compression, approximately equal compression and relaxation times should be used, and interruptions in chest compressions should be minimized.

LUCAS™ (LUCAS V2US; Jolife AB, Lund, Sweden) is a CPR device providing automatic 5 cm deep compressions and active decompressions back to normal position with a frequency of 100 per minute, and a duty cycle (compression time) of 50%, i.e., LUCAS is adjusted to give chest compressions according to the latest guidelines. LUCAS was introduced in clinical practice in Sweden by Steen and coworkers in the year 2000, and the first scientific report on its properties, based on 100 pigs and 20 patients, was published in 2002 [14]. Since then several experimental and clinical studies on LUCAS have been published, which confirms its efficacy in supporting quality chest compressions [14-18].

No comparison between mechanical and manual compression performed according to the 2005 guidelines has been made in an experimental study. Our hypothesis is that mechanical compression/decompression will give higher coronary perfusion pressure in a model with prolonged CPR, simulating ventricular fibrillation resistant to defibrillation. It is well known that external chest compressions are associated with rib fractures. LUCAS CPR gives a standardized compression depth of maximum 5 cm, whereas with manual CPR there is no control regarding the compression depth. Therefore a second hypothesis was that less rib fractures would occur in the LUCAS-CPR group.

## Methods

**Figure 1:**
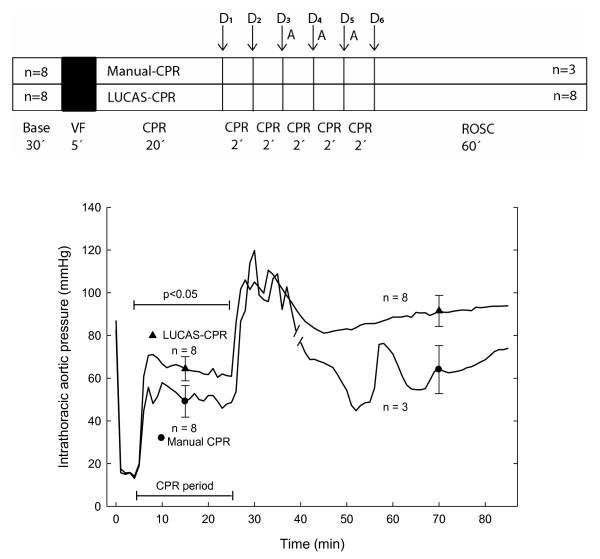
**The design of the study (upper panel)**. The number of pigs with return of spontaneous circulation (ROSC) is indicated within the ROSC rectangle. VF: ventricular fibrillation; CPR: cardiopulmonary resuscitation; D_1_-D_6_: defibrillations; A: adrenaline 0.01 mg/kg given intravenously. The lower panel shows the mean systolic pressure and during CPR the compression pressure, in the intrathoracic aorta during the experiment. The CPR period is marked. The break in the manual CPR curve marks where n is changed from 8 to 3 individuals. Mean ± SEM is included in 2 places in each curve; n = 8, except for the ROSC period for manual CPR, where n = 3.

We have described in detail our pig model for CPR research elsewhere [14]. The design of the experiment is shown in Figure 1. Swedish domestic pigs were stratified into one manual CPR group (n = 8, mean weight 31 kg, range 29-33 kg) and one LUCAS-CPR group (n = 8, mean weight 31 kg, range 28-32 kg). The chest compressions were performed with the pigs in a supine position. All pigs received humane care in compliance with the European Convention for the Protection of Vertebrate Animals used for Experimental and Other Scientific Purposes (1986) and the 'Guide for the Care and Use of Laboratory Animals' published by the National Institutes of Health (NIH Publication 85-23, revised 1985). The study was approved by the ethics committee for animal experiments at Lund University, Sweden.

### Anesthesia and ventilation

Anesthesia was induced with an intramuscular injection of ketamine (30 mg/kg) and xylasin (4 mg/kg). Sodium thiopental (5-8 mg/kg) and atropine (0.015 mg/kg) were given intravenously before tracheotomy. Anesthesia and muscular paralysis were maintained with a continuous infusion of 10 ml/h of a NaCl (0.9%) solution containing ketamine (16 mg/ml) and pancuronium (0.6 mg/ml).

A Boussignac ET tube for cardiac arrest (Laboratories Pharmaceutiques VYGON, Ecouen, France, 7 mm internal diameter) was used as an ordinary endotracheal tube. Intratracheal pressure was measured through one of the distal lines incorporated in the wall of the Boussignac ET tube. The tube was connected to a Servo Ventilator 300 (Servo Ventilator 300, Siemens, Solna, Sweden) using pressure-regulated (max 30 cmH_2_O = 23 mmHg) and volume-controlled intermittent positive pressure ventilation (IPPV). Normo-ventilation (end-tidal CO_2 _around 5.3 kPa = 40 mmHg) was obtained by using a tidal volume of 8 ml/kg body weight, 20 breaths/min, a PEEP of 5 cmH_2_O and a FiO_2 _of 0.21.

During CPR ventilation was given, without interruption of chest compressions, by means of a Ruben bag in both the manual CPR and the LUCAS-CPR group. Ventilation was given with 100% oxygen, the frequency was 1 ventilation after every 10th compression (= 10 ventilations/min) in both groups. After return of spontaneous circulation (ROSC) the animals in both groups were given baseline ventilation by the Servo Ventilator 300 with a FiO_2 _of 1.0.

### Preparation and monitoring

Four catheters for blood pressure measurements and blood sampling were introduced into the right carotid artery and the right internal jugular vein. The catheters were inserted into the ascending aorta and into the right atrium, respectively. An ultrasonic flow probe connected to a MediStim flowmeter apparatus (CM-4000, MediStim ASA, Oslo, Norway) was placed on the left internal carotid artery. A temperature probe was inserted into the esophagus. Three-lead electrocardiogram (ECG) were obtained by electrodes corresponding to R,L,F and ground were adhered to the skin.

### Chest compressions

VF was induced with a 5-20 mA and 6-14 Hz square formed wave current delivered to the epicardial surface via a needle electrode puncture through the upper abdomen. CPR was started 5 minutes after induction of VF.

In the manual CPR experiments, 16 paramedics and ambulance nurses from Lund Ambulance Station, 10 men and 8 women (mean length 175 cm, range 162-188 cm, mean weight 76 kg, range 55-95 kg) were responsible for the chest compressions and ventilations. Two paramedics carried out the compressions at each manual CPR experiment and they shifted between doing chest compressions and ventilation every second minute. A sound indicator was used to keep the frequency of the manual compressions at 100/min. Within 14 days before the experiments were done, all rescuers had to undergo manual CPR training on a mannequin and were instructed to give chest compressions according the international guidelines from 2005. The exact spot for delivery of the manual chest compressions were decided by placing of LUCAS on all pigs in both groups and mark the optimal compressions spot by drawing an ink line around the suction cup. During the 5 minutes with VF, LUCAS was placed in correct position so that the compressions could start at scheduled time. The compressions were given on sternum between the inferior 1/3 and superior 2/3 of sternum. The marked place was used for both manual and mechanical chest compressions. The anterior-posterior diameter of the thorax was measured by means of a ruler before and after CPR.

LUCAS (V2US; Jolife AB), also used in humans, was used to deliver the mechanical chest compressions. According to an international agreement (Utstein-style guidelines for uniform reporting of laboratory CPR research. Resuscitation 1996;33:69-84) 20-25 kg pigs are recommended to use as they have similar anterior-posterior diameter as adult humans.

### Return of spontaneous circulation

Defibrillations, if indicated, were done externally using biphasic shocks (Lifepak 12, Medtronic, Minneapolis, MN, USA), using 360 J energy through pads (see Figure 1). Between each shock, chest compressions for 2 minutes were to be given. At manual CPR there was to be minimal delay (around 2 seconds) between chest compression and defibrillation. With LUCAS-CPR, defibrillation was given during ongoing CPR. The research leader judged whether ROSC had been obtained after each defibrillation (systolic arterial pressure above 60 mmHg for 1 minute). How to give defibrillation and adrenaline was adjusted to the experimental situation. If ROSC was not obtained after 3 defibrillations, adrenaline 0.01 mg/kg was given in the central venous catheter 3 times, with 2-minute intervals of chest compressions between each dose. After the third dose of adrenaline, CPR was continued for 2 minutes and then terminated. If ROSC was obtained, measurements continued for 1 hour.

### Autopsy

After terminating each experiment an autopsy was performed. For obvious reasons (suction marks on the skin) could the autopsy not be made blinded. The position of catheters and the presence of any fractures in ribs or sternum, or injuries on lungs, heart and abdominal organs were noted.

### Calculation of the coronary perfusion pressure

**Figure 2:**
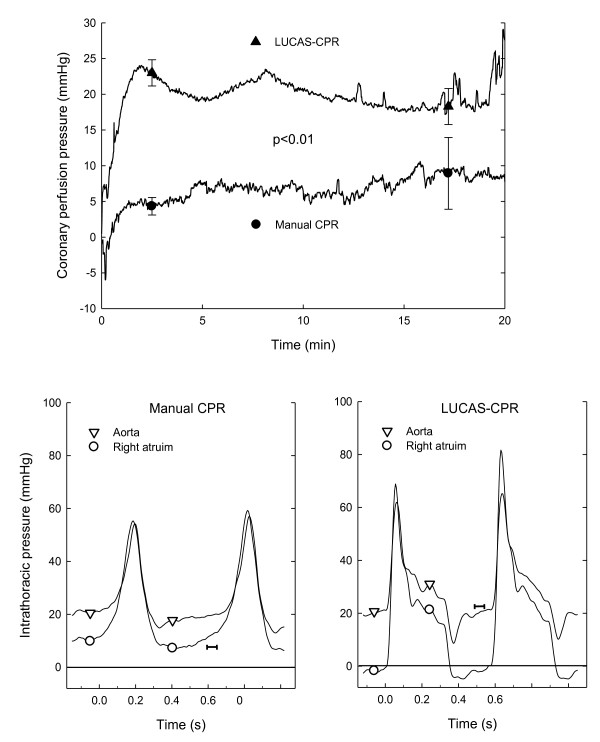
**The coronary perfusion pressure in the two groups (upper panel)**. The lower panels show typical pressure curves in the intrathoracic aorta (triangles) and the right atrium (circles) during two cycles of CPR in the manual CPR (left) and LUCAS-CPR (right) groups just after a ventilation. A bar (|--|) is inserted before one compression in both panels. The 0.05-second long bar shows where in the cycle CPP is calculated (as the difference between the pressure in aorta and right atrium). The level of the bar shows the CPP in this registration; 7 mmHg in the manual group and 22 mmHg in the LUCAS group.

The coronary perfusion pressure (CPP) was computed as the difference between the intrathoracic aortic and the right atrial pressure in the end-decompression phase. The beginning of each compression cycle was defined as the maximal pressure rise ratio (see Figure 2). The end-decompression phase was measured in a window between 0.1 to 0.05 seconds before the compression. The mean of the sampled values during that window of 0.05 seconds (10 values, i.e., 200 Hz) was calculated.

#### Statistics

A sample size of 8 in each group was used. By use of the following parameters sample size calculation showed that n had to be at least n = 7 in each group. 1: A standard deviation of 4.9 mmHg for the measurement of CPP during manual compressions as found in an earlier study [14]. 2: A difference of 7 mmHg between groups. 3: A significance level of 5%.

Global interpretation of data was done by means of the area under curve comparing different variables between the two groups during the CPR period. Student's t-test for unpaired observations was used. Data obtained during the first 90 seconds of the CPR period was excluded from the statistical calculations because this is the time required to reach plateau. Fisher's Exact Test was used for autopsy findings. A p-value <0.05 was regarded as indicative of a statistically significant difference between the groups.

## Results

### ROSC

In the manual group five pigs did not achieve ROSC. One pig obtained ROSC after 1 defibrillation, 1 pig obtained ROSC after 1 defibrillation followed by 2 minutes of manual CPR and 1 pig obtained ROSC after 1 defibrillation followed by 10 minutes of manual CPR and intravenous adrenaline 0.01 mg/kg 3 times at 2-minute intervals, according to the protocol (Figure 1).

In the LUCAS group all 8 animals obtained ROSC. In 5 pigs, ROSC was obtained after the first defibrillation. In 2 pigs ROSC was obtained after one defibrillation followed by 2 minutes of LUCAS-CPR. In 1 pig, ROSC was obtained after 1 defibrillation, 4 minutes of LUCAS-CPR and the first dose of 0.01 mg/kg of intravenous adrenaline according to the protocol (Figure 1).

### Number of compressions given during the 20-minute CPR-period

About two thousand compressions were given to each animal in the LUCAS group. The mean time for the paramedics to change between compression and ventilation was 4 ± 1 seconds, i.e., each pig received about 60 compressions less in the manual group.

### Aortic pressure during compression phase

The aortic compression pressure in the LUCAS-group was around 65 mmHg, and in the manual group around 55 mmHg during the CPR period (p < 0.05)(Figure 1).

### Intrathoracic aortic and right atrial pressures during decompression phase

The intrathoracic aortic pressure during the decompression phase varied between 5 and 15 mmHg in the manual group and was significantly higher in the LUCAS group where it varied between 10 and 25 mmHg (p < 0.05). The right atrial pressure during the decompression phase was between 5 and 10 mmHg in the manual group whereas it was significantly lower in the LUCAS group, where it varied between -5 and 5 mmHg (p < 0.01).

### Coronary perfusion pressure

The coronary perfusion pressure was between 20 and 25 mmHg in the LUCAS group during CPR which was significantly (p < 0.01) higher than in the manual group where it was between 5 and 10 mmHg (Figure 2). There was no overlap of CPP between the two groups.

### Left carotid artery flow

**Figure 3:**
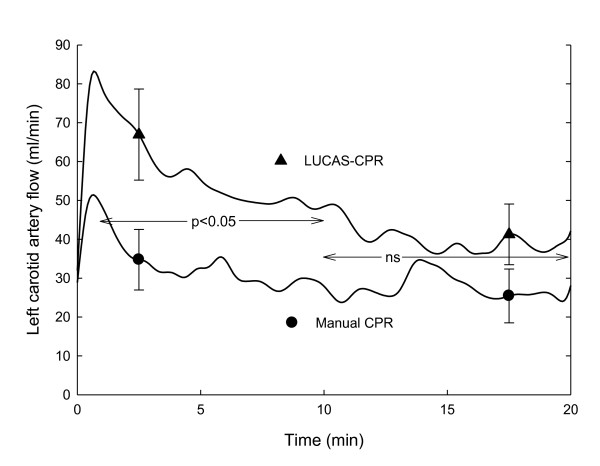
**Left carotid artery blood flow in the manual CPR and LUCAS-CPR groups during the CPR period**. Statistically significant differences between the groups are shown. Mean ± SEM, n = 8 in both groups.

The mean flow at baseline was 176 ± 17 ml/min in the manual group and 212 ± 33 ml/min in the LUCAS group (not significant). During CPR, the flow was significantly higher (p < 0.05) in the LUCAS-CPR group during the first 10 minutes (Figure 3).

### Electrocardiogram

All ECG recordings just before initiation of ventricular fibrillation showed sinus rhythm with a mean rate of 95/min (n = 16).

In the LUCAS group there was no sign of ischemia 1 hour after ROSC; in the 3 animals with ROSC in the manual group, ECG was also normal, except in one pig where negative T-waves were seen.

### End-tidal CO_2_

The ETCO_2_-values were around 3.4 kPa in the LUCAS group and around 2.2 kPa in the manual group; this difference was statistically significant (p < 0.05).

### Peak airway pressure

The peak airway pressure varied between 15 to 25 mmHg, and there was no statistically significant difference between the groups.

### Blood gases

There were no significant differences in blood gases (Table 1). The arterial oxygenation was excellent in both groups. The animals in both groups had subnormal PaCO_2_-values during CPR, i.e., the animals were slightly hyperventilated (Table 1).

**Table 1:** Blood gases at baseline, during CPR, and during ROSC in the manual group and the LUCAS group.

	Baseline	20 min CPR	60 min ROSC
**Temperature (°C)**			
Manual	37.1 ± 0.4	36.8 ± 0.3	35.8 ± 0.5
LUCAS	37.5 ± 0.2	36.6 ± 0.3	36.4 ± 0.3
			
**Hbv (g/l)**			
Manual	111 ± 2	127 ± 3	109 ± 6
LUCAS	109 ± 2	120 ± 2	110 ± 2
			
**Hctv (%)**			
Manual	34 ± 1	39 ± 1	33 ± 2
LUCAS	34 ± 1	37 ± 1	34 ± 1
			
**SaO**_**2 **_**(%)**			
Manual	92 ± 0	95 ± 1	95 ± 1
LUCAS	91 ± 0	94 ± 0	95 ± 0
			
**SvO**_**2 **_**(%)**			
Manual	66 ± 3	26 ± 3	50 ± 9
LUCAS	66 ± 2	24 ± 2	61 ± 5
			
**PaO**_**2 **_**(kPa)**			
Manual	13.4 ± 0.3	53.5 ± 4.5	71.1 ± 3.3
LUCAS	12.6 ± 0.3	54.6 ± 2.7	62.4 ± 2.7
			
**PvO**_**2 **_**(kPa)**			
Manual	6.0 ± 0.3	2.9 ± 0.2	5.0 ± 0.3
LUCAS	5.9 ± 0.2	3.3 ± 0.6	5.7 ± 3.3
			
**PaCO**_**2 **_**(kPa)**			
Manual	5.5 ± 0.3	3.1 ± 0.4	5.6 ± 0.9
LUCAS	5.6 ± 0.3	4.0 ± 0.4	5.6 ± 0.3
			
**PvCO**_**2 **_**(kPa)**			
Manual	6.6 ± 0.4	8.0 ± 0.6	8.8 ± 1.6
LUCAS	6.5 ± 0.3	8.1 ± 0.5	7.7 ± 0.6
			
**pHa**			
Manual	7.48 ± 0.01	7.47 ± 0.03	7.38 ± 0.06
LUCAS	7.47 ± 0.01	7.42 ± 0.02	7.42 ± 0.02
			
**pHv**			
Manual	7.42 ± 0.01	7.27 ± 0.02	7.24 ± 0.07
LUCAS	7.43 ± 0.01	7.25 ± 0.01	7.32 ± 0.03
			
**Base excess a (mmol/l)**			
Manual	6.2 ± 0.7	-5.5 ± 0.7	-0.4 ± 3.6
LUCAS	6.4 ± 0.7	-4.4 ± 0.8	2.4 ± 1.1
			
**Base excess v (mmol/l)**			
Manual	6.5 ± 0.8	-1.4 ± 0.6	-0.9 ± 3.7
LUCAS	6.5 ± 0.7	-2.2 ± 0.7	2.1 ± 1.2
			
**Lactate v (mmol/l)**			
Manual	1.5 ± 0.2	6.1 ± 0.2	6.6 ± 1.9
LUCAS	1.5 ± 0.2	6.3 ± 0.2	4.3 ± 0.5
			
**Glucose v (mmol/l)**			
Manual	5.6 ± 0.4	15.0 ± 1.5	11.8 ± 3.1
LUCAS	5.9 ± 0.4	14.7 ± 0.7	11.6 ± 1.3

N = 8, except at 60 min ROSC in the manual group were n = 3.

Hbv = hemoglobin in venous blood; Hctv = hematocrite in venous blood; SaO_2 _and SvO_2 _= oxygen saturation in arterial and venous blood, respectively; PaO_2 _and PvO_2 _= partial oxygen pressure in arterial and venous blood, respectively; PaCO_2 _and PvCO_2 _= partial carbon dioxide pressure in arterial and venous blood, respectively; pHa and pHv = pH in arterial and venous blood, respectively; base excess a and base excess v = base excess in arterial and venous blood, respectively; lactate v and glucose v = lactate and glucose in venous blood. Blood gases are given at temperature-corrected values.

### Flattening of thorax after CPR

The anterior-posterior diameter decreased by 25 ± 2 mm in the manual group and by 20 ± 2 mm in the LUCAS group, a difference of 5 mm (p = 0.14)

### Autopsy

In the 5 pigs that did not achieve ROSC in the manual group, the hearts were in ischemic contracture (stone heart) with no lumen seen on cross section of the left ventricle. In the manual group there was significantly (p < 0.01) more rib fractures; manual group left side 33, right side 21, and LUCAS group left side 30, right side 3. Two serious injuries were seen in the manual group, one right-sided pressure pneumothorax (air escaping when the right pleura was opened, right lung collapsed), and one vertical deep liver rupture (500 ml blood could be sucked out from the abdomen, which is about 20% of the blood volume of a pig this size).

## Discussion

In the porcine model used in the present study, LUCAS-CPR was more efficient and caused less trauma than manual CPR. The shape of the pig thorax is different from the human thorax [14] and therefore the results obtained in the manual group should be interpreted with caution. The human thorax in the supine position is like an egg laying on its side whereas in the same position the pig thorax is like an egg standing on its end. In pigs, the ventricles are positioned in the center of the thoracic cavity, surrounded by lung tissue on all sides. In humans, the right ventricle is positioned just under the sternum. This anatomic difference makes it more difficult to get a compression effect on the heart in pigs where the compressions affect the heart only by 'the thoracic pump mechanism', e.g., a chest compression increases the intrathoracic pressure which in turn affects the heart. In humans not only 'the thoracic pump mechanism' but also a 'heart pump mechanism' works, i.e. a direct compression of the heart by a chest compression. Patients with chronic obstructive pulmonary disease (COPD) have a thorax that is more like the porcine thorax with lungs surrounding the heart on all sides.

The animals in the LUCAS-group received continuous compressions without a need to stop due to rescuer fatigue, or for rescuer safety during defibrillation attempts. Therefore the animals in the manual group received about 60 compressions less than those in the LUCAS-group. For each change between ventilation and compression, the animals were without CPR for about 4 seconds, during which time the coronary perfusion pressure dropped to zero, and when the compressions were started again, it took about 10 seconds to regain the CPP that had been obtained during the previous 2-minute period of continuous manual CPR.

Studies have shown the difficulty in giving optimal manual compressions consistently without fail, many compressions are either too shallow [6] or too deep [4]. Too deep compressions may cause severe injuries, as was seen in one pig in the manual group. There were significantly more rib fractures in the manual group; typically, in the manual group there were rib fractures on both sides, whereas in the LUCAS-group there were fractures only on one side. After the experiment, the thorax recoiled better in the LUCAS-group, because most ribs were intact on one side. The anterior-posterior diameter of the chest at the point where the compressions were given was on average 5 mm less in the manual group based on measurements before and after compressions. This difference between groups was not statistically significant, but co-incided with a visibly flatter appearance of the anterior part of the chest in some of the animals in the manual group. In a clinical CPR study comparing LUCAS with manual chest compressions, Smekal et. al. showed no increased rate of injuries in deceased victims in the LUCAS CPR group [19]. Though, patients allotted to a mechanical chest compression group get manual chest compression initially before mounting the device. This makes it impossible to compare the true difference between mechanical and manual chest compression in clinical studies.

The suction cup of the LUCAS device provides active decompression of the chest. This resulted in a negative pressure in the right atrium during the initial part of decompression phase (Figure 2). The right atrial pressure during the decompression phase was between 5 and 10 mmHg in the manual group whereas in the LUCAS group it was between -5 and 5 mmHg, i.e., it was significantly lower in the LUCAS group. This, together with a significantly higher intrathoracic aortic pressure during the end-decompression phase in the LUCAS group, explains the significantly higher CPP in that group. Paradise and co-workers found that only patients with a coronary perfusion pressure of 15 mmHg or higher got ROSC [20]. In an earlier study we have found the same for pigs [14]. A CPP of only 5 mmHg, as seen in the manual group, probably explains the lower ROSC rate. Another possible explanation for the higher ROSC rate in the LUCAS CPR group was that they were defibrillated during ongoing compressions [15]. However, in the present study the delay between compressions and defibrillation was only 2 seconds, which is too short for a significant drop in CPP. Therefore, we think that the lower ROSC rate in the manual group was caused by the low CPP in this model with prolonged CPR.

Rubertsson and Karlsten [16] used a device (Cardiopress) for standardized manual chest compressions and compared it with LUCAS in a porcine model. During CPR mean cortical cerebral blood flow in the group treated with LUCAS compressions reached a level of approximately 65% of baseline blood flow that was stable throughout the whole CPR period. In the manual group the mean cortical cerebral blood flow was statistically significantly lower, around 40%.

If the ventilation is standardized during CPR, as in the present study, end tidal CO_2 _can be used as an index of the blood flow through the lungs [21]. End tidal CO_2 _was significantly higher in the LUCAS group, indicating a higher blood flow in that group.

## Conclusions

LUCAS-CPR is significantly more efficient and gives less injury than manual CPR in this porcine model.

## Competing interests

Jolife AB has given economical support for the research in cardiopulmonary resuscitation done by Stig Steen. There are no other competing financial or non-financial interests.

## Authors' contributions

All authors participated in the design of the study and performed the experiments. QL, TS and SS drafted the manuscript. All authors read and approved the final manuscript.

## Pre-publication history

The pre-publication history for this paper can be accessed here:

http://www.biomedcentral.com/1471-2261/10/53/prepub

## References

[B1] Hightower D Thomas SH Stone CK Dunn K March JA Decay in quality of closed-chest compressions over time Ann Emerg Med 1995 26 300 303 10.1016/S0196-0644(95)70076-5 7661418

[B2] Sunde L Wik K Steen PA Quality of mechanical, manual standard and active compression-decompression CPR on the arrest site and during transport in a manikin model Resuscitation 1997 34 235 242 10.1016/S0300-9572(96)01087-8 9178384

[B3] Ocha FJ Ramalle-Gómara E Lisa V Saralegui I The effect of rescuer fatigue on the quality of chest compressions Resuscitation 1998 37 149 152 10.1016/S0300-9572(98)00057-4 9715774

[B4] Thorén AB Axelsson A Holmberg S Herlitz J Measurement of skills in cardiopulmonary resuscitation - do professionals follow given guidelines? Eur J Emerg Med 2001 8 169 176 10.1097/00063110-200109000-00002 11587460

[B5] Ashton A McCluskey A Gwinnutt CL Keenan AM Effect of rescuer fatigue on performance of continuous external chest compressions over 3 min Resuscitation 2002 55 151 155 10.1016/S0300-9572(02)00168-5 12413752

[B6] Wik L Kramer-Johansen J Myklebust H Sørebø H Svensson L Fellows B Steen PA Quality of cardiopulmonary resuscitation during out-of-hospital cardiac arrest JAMA 2005 293 299 304 10.1001/jama.293.3.299 15657322

[B7] Heidenreich JW Berg RA Higdon TA Ewy GA Kern KB Sanders AB Rescuer fatigue: standard versus continuous chest-compression cardiopulmonary resuscitation Acad Emerg Med 2006 13 1020 1026 10.1111/j.1553-2712.2006.tb00272.x 17015418

[B8] Aufderheide TP Pirrallo RG Yannopoulos D Klein JP von C Sparks CW Deja KA Kitscha DJ Provo TA Lurie KG Incomplete chest wall decompression: a clinical evaluation of CPR performance by trained laypersons and an assessment of alternative manual chest compression-decompression techniques Resuscitation 2006 71 341 351 10.1016/j.resuscitation.2006.03.021 17070644

[B9] Kim JA Vogel D Guimond G Hostler D Wang HE Menegazzi JJ A randomized, controlled comparison of cardiopulmonary resuscitation performed on the floor and on a moving ambulance stretcher Prehosp Emerg Care 2006 10 68 70 10.1080/10903120500373108 16418093

[B10] Edelson DP Abella BS Kramer-Johansen J Wik L Myklebust H Barry AM Merchant RM Hoek TL Steen PA Becker LB Effect of compression depth and pre-shock pauses predict defibrillation failure during cardiac arrest Resuscitation 2006 71 137 145 10.1016/j.resuscitation.2006.04.008 16982127

[B11] Olasveegen TM Tomlison AE Wik L Sunde K Steen PA Myklbust H Kramer-Johansen J A failed attempt to improve quality of out-of-hospital CPR through performance evaluation Prehosp Emerg Care 2007 11 427 433 10.1080/10903120701536628 17907028

[B12] European Resuscitation Council Guidelines 2005 Resuscitation 2005 67S1

[B13] 2005 American Heart Association Guidelines for Cardiopulmonary Resuscitation and Emergency Cardiovascular Care Circ 2005 112 10.1161/CIRCULATIONAHA.105.166550 16314375

[B14] Steen S Liao Q Pierre L Paskevicius A Sjöberg T Evaluation of LUCAS, a new device for automatic mechanical compression and decompression resuscitation Resuscitation 2002 55 285 299 10.1016/S0300-9572(02)00271-X 12458066

[B15] Steen S Liao Q Pierre L Paskevicius A Sjöberg T The critical importance of minimal delay between chest compressions and subsequent defibrillation: a haemodynamic explantation Resuscitation 2003 58 249 258 10.1016/S0300-9572(03)00265-X 12969599

[B16] Rubertsson S Karlsten R Increased cortical cerebral blood flow with LUCAS, a new device for mechanical chest compressions compared to standard external compressions during experimental cardiopulmonary resuscitation Resuscitation 2005 65 357 363 10.1016/j.resuscitation.2004.12.006 15919574

[B17] Steen S Sjöberg T Olsson P Young M Treatment of out-of-hospital cardiac arrest with LUCAS, a new device for automatic mechanical compression and active decompression resuscitation Resuscitation 2005 67 25 30 10.1016/j.resuscitation.2005.05.013 16159692

[B18] Nielsen N Sandhall L Scherstén F Friberg H Olsson SE Successful resuscitation with mechanical CPR, therapeutic hypothermia and coronary intervention during manual CPR after out-of-hospital cardiac arrest Resuscitation 2005 65 111 3 10.1016/j.resuscitation.2004.11.007 15797284

[B19] Smekal D Johansson J Huzrvka T Rubertsson S No difference in autopsy detected injuries in cardiac arrest patients treated with manual chest compared with mechanical compressions with the LUCAS TM device - A pilot study Resuscitation 2009 80 1104 7 10.1016/j.resuscitation.2009.06.010 19595496

[B20] Paradis NA Martin GB Rivers EP Coronary perfusion pressure and the return of spontaneous circulation in human cardiopulmonary resuscitation JAMA 1990 263 1106 1113 10.1001/jama.263.8.1106 2386557

[B21] Levine RL Wayne MA Miller CC End-tidal carbon dioxide and outcome of out-of-hospital cardiac arrest N Engl J Med 1997 337 301 306 10.1056/NEJM199707313370503 9233867

